# NAFLD as a continuum: from obesity to metabolic syndrome and diabetes

**DOI:** 10.1186/s13098-020-00570-y

**Published:** 2020-07-14

**Authors:** Amélio F. Godoy-Matos, Wellington S. Silva Júnior, Cynthia M. Valerio

**Affiliations:** 1 Metabolism Department, Instituto Estadual de Diabetes e Endocrinologia (IEDE), Pontifical Catholic University of Rio de Janeiro (PUC-Rio), Rio de Janeiro, RJ CEP 20211-340 Brazil; 2 Endocrinology Discipline, Faculty of Medicine, Center of Natural, Human, Health, and Technology Sciences, Federal University of Maranhão (UFMA), Pinheiro, MA CEP 65200-000 Brazil

**Keywords:** NAFLD, NASH, Fatty liver, Obesity, Metabolic syndrome, Diabetes

## Abstract

**Background:**

The prevalence of non-alcoholic fatty liver disease (NAFLD) has been increasing rapidly. It is nowadays recognized as the most frequent liver disease, affecting a quarter of global population and regularly coexisting with metabolic disorders such as type 2 diabetes, hypertension, obesity, and cardiovascular disease. In a more simplistic view, NAFLD could be defined as an increase in liver fat content, in the absence of secondary cause of steatosis. In fact, the clinical onset of the disease is a much more complex process, closely related to insulin resistance, limited expandability and dysfunctionality of adipose tissue. A fatty liver is a main driver for a new recognized liver-pancreatic α-cell axis and increased glucagon, contributing to diabetes pathophysiology.

**Main text:**

This review will focus on the clinical and pathophysiological connections between NAFLD, insulin resistance and type 2 diabetes. We reviewed non-invasive methods and several scoring systems for estimative of steatosis and fibrosis, proposing a multistep process for NAFLD evaluation. We will also discuss treatment options with a more comprehensive view, focusing on the current available therapies for obesity and/or type 2 diabetes that impact each stage of NAFLD.

**Conclusion:**

The proper understanding of NAFLD spectrum—as a continuum from obesity to metabolic syndrome and diabetes—may contribute to the early identification and for establishment of targeted treatment.

## Background

Nonalcoholic fatty liver disease (NAFLD) is a spectrum of hepatic diseases associated with metabolic and cardiovascular disorders, such as obesity, insulin resistance (IR), hypertension, dyslipidemia and type 2 diabetes (T2D). It is frequently recognized as the hepatic manifestation of the metabolic syndrome (MetS) [1] and constitute the most frequent liver condition worldwide [2–4].

NAFLD is characterized by increased liver fat content, with a threshold of > 5%, in the absence of significant alcohol consumption or other secondary cause of steatosis, including alcohol consumption (characterized as 30 g/day for men and 20 g/day for women) [5, 6]. It can be subcategorized as nonalcoholic fatty liver (NAFL), when there is only evidence of hepatic steatosis on liver histology, and nonalcoholic steatohepatitis (NASH), when there are steatosis, lobular inflammation and hepatocyte ballooning with or without perisinusoidal fibrosis [3]. NAFLD may progress to cirrhosis and hepatocellular carcinoma, but its cardiometabolic counterparts are the main cause of morbimortality in those patients [4, 7].

A panel of 22 international experts recently proposed the definition criteria for the metabolic-associated fatty liver disease (MAFLD) [8]. MAFLD is defined as the presence of hepatic steatosis (histological, imaging or blood biomarker evidence of hepatic steatosis) plus at least one of three metabolic criteria: overweight/obesity, established T2D or the presence of metabolic dysregulation [8]. The latter is characterized by the presence of at least 2 metabolic abnormalities (Table 1).

**Table 1:** Criteria defining metabolic dysregulation in the context of metabolic-associated fatty liver disease. Adapted from [8]

Any two of the seven criteria below:
WC ≥ 102/88 cm (Caucasian men and women) or ≥ 90/80 cm (Asian men and women)
HDL cholesterol < 40 mg/dL (1.0 mmol/L) in men, < 50 mg/dL (1.3 mmol/L) in women or specific drug treatment
Plasma triglycerides > 150 mg/dL (1.7 mmol/L) or specific drug treatment
Blood pressure > 130/85 mmHg or specific drug treatment
Prediabetes
HOMA-IR score ≥ 2.5
hsCRP level > 2 mg/L

*HDL* high-density lipoprotein, *HOMA-IR* homeostasis model assessment of insulin resistance, *hsCRP* high-sensitivity C-reactive protein level, *WC* waist circumference

Importantly, this “MAFLD definition” avoid the dichotomous view of NAFL and NASH, since it is based in “positive” criterion (evidence of hepatic steatosis) instead of “negative” criterion hard to exclude (i.e., alcohol ingestion quantification), and also allows concomitant dual etiology or “alternate causes” (e.g., alcohol, medications or rare diseases) in association with a metabolic risk profile [8].

Therefore, the aiming of this article is to review epidemiology, pathophysiology, diagnosis and treatment of NAFLD with focus on its metabolic profile and evolution through the natural history of obesity, MetS and T2D.

## Epidemiology

Although epidemiological data involving more than 8 million people estimated a global prevalence of NAFLD around 25% [2], it certainly varies greatly depending on how it is diagnosed and on the region of the world considered. Importantly, the 2 highest regional prevalence were observed in Middle East and South America (approximately 30%) [2]. Roughly 60% of those people subjected to liver biopsy presented with NASH. In accordance with its metabolic nature, 42% of NAFLD subjects had MetS; 69%, hyperlipidemia; 51%, obesity; 39%, hypertension; and 22%, diabetes [2].

### Obesity

The prevalence of NAFLD increases in parallel with the increasing prevalence in obesity, MetS and T2D. The number of people with obesity have increased globally from 1975 to 2014, when 11% of adult men and 15% of adult women were diagnosed with this condition [9]. In Brazil, obesity increased 67.8% within 13 years, reaching 19.8% in 2018 [10].

As introduced above, worldwide prevalence of obesity among NAFLD and NASH patients were 51 and 81%, respectively [2]. In populations with obesity, NAFLD prevalence varies from 60 to 95% [11, 12].

Fat distribution is a main pathophysiological mechanism for metabolic disease, and abdominal obesity may differ from a more equally fat distribution. Although a recent consensus underscores the importance of measuring waist circumference (WC) as part of a more reliable estimate of metabolic risk, abdominal obesity prevalence has increased more than general obesity by a given body mass index (BMI) [13]. Additionally, in a cohort of 2017 subjects followed-up for 4.4 years, visceral fat area, as estimated by ultrasonography (US) or computed tomography (CT), was longitudinally associated with incidence of NAFLD, with an adjusted hazard ratio of 2.23 (95% CI 1.28–3.89) [14].

### Metabolic syndrome

MetS is characterized as a cluster of metabolic disorders such as abdominal obesity, hypertension, dyslipidemia and impaired glycemia [15]. It has 2 mains definitions (Table 2) and is highly prevalent worldwide [16, 17]. According to the National Health and Nutrition Examination Survey (NHANES), more than a third of American adults presented MetS, with an increment of more than 35% from 1988–1994 to 2007–2012 [18, 19]. Comprehensively, as obesity rate rises, so does the prevalence of MetS. In ten large European cohorts (163,517 individuals), the age-standardized percentage of obese subjects with MetS ranged from 24 to 65% in women and from 43% to 78% in men [20].

**Table 2:** Main definitions of metabolic syndrome. Adapted from [16, 17]

Adult Treatment Panel III (2005 revision)	International Diabetes Federation
Any three of the five criteria below:	WC ≥ 94 cm (men) or ≥ 80 cm (women) and at least two of the following:
WC > 102 cm (men) or > 88 cm (women)	Blood glucose > 100 mg/dL (5.6 mmol/L) or diagnosed diabetes
Blood glucose > 100 mg/dL (5.6 mmol/L) or diagnosed diabetes	HDL cholesterol < 40 mg/dL (1.0 mmol/L) in men, < 50 mg/dL (1.3 mmol/L) in women or specific drug treatment
HDL cholesterol < 40 mg/dL (1.0 mmol/L) in men, < 50 mg/dL (1.3 mmol/L) in women or specific drug treatment	Plasma triglycerides > 150 mg/dL (1.7 mmol/L) or specific drug treatment
Plasma triglycerides > 150 mg/dL (1.7 mmol/L) or specific drug treatment	Blood pressure > 130/85 mmHg or specific drug treatment
Blood pressure > 130/85 mmHg or specific drug treatment	

*HDL* high-density lipoprotein, *WC* waist circumference

The association of MetS with the prevalence and severity of NAFLD, assessed by US and NAFLD Fibrosis score (NFS), was evaluated in a cohort of 11,647 individuals [21]. Despite the prevalence of NAFLD was 18.2% (95% CI 16.5–19.9), it was significantly greater (43.2%) in those with MetS (OR 11.5, 95%CI 8.9–14.7) and increased with the number of MetS criteria (67% for those with all five criteria). More important, advanced hepatic fibrosis was present in 6.6% in those with moderate/severe steatosis, almost doubled in the presence of MetS and reached impressive 30% in those with five MetS criteria [21].

### Diabetes

Diabetes is one of the fastest growing global health emergencies of the 21st century [22]. Around 463 million people worldwide was living with diabetes in 2019, and a 51% increase is expected to 2045, raising the prevalence of diabetes to 700 million. Brazil is the fifth country with the highest number of people with diabetes in the world (16.8 million) [22].

The association between T2D and NAFLD is well established. Notwithstanding, physicians may not be conscious enough how this association may be deleterious [23]. NAFLD is highly prevalent in T2D patients, according to two meta-analyses [24, 25]. Dai et al. [24] extracted data from 24 studies with 35,599 T2D patients and found a pooled NAFLD prevalence of 59.67% (95% CI 54.31–64.92), which rose to 77.87% (95% CI 65.51–88.14) in those with obesity. Moreover, data from 80 studies (49,419 individuals) evidenced a global NAFLD prevalence of 55.5% (95% CI 47.3–63.7) among patients with T2D [25]. Pooled studies carried out in Europe evidenced 68% (95% CI 62.1–73.0) of prevalence, which was the highest globally. The estimated prevalence of NASH and advanced fibrosis among individuals with NAFLD and T2D were 37.3% (95% CI 24.7–50.0) and 17.0% (95% CI 7.2–34.8), respectively [25]. Furthermore, the overall mortality ratio in 5–10 years was 585 per 100,000, which was greater than mortality from others chronic liver diseases. The majority of the NAFLD patients with T2D fulfilled criteria for MetS, highlighting the relationship between these conditions in the metabolic risk continuum.

## Pathogenesis

Overweight and obesity are the main drivers of metabolic diseases and NAFLD. Nevertheless, not all obese are metabolically unhealthy, neither all normal weight/lean are metabolically healthy. Fat distribution, adipose tissue (AT) functionality and IR constitute the basis of metabolic disturbances such as MetS, diabetes and NAFLD [26].

More than 10 years ago, Virtue and Puig [27] putted forward the “AT expandability hypothesis”, by which the capacity for stock lipids by expanding AT is limited in an individualized fashion. Therefore, when the capacity of expansion is reached, lipids can no longer be stored in AT and it is stored in ectopic tissues, like muscle and liver, where promotes IR, through a lipotoxic effect.

The AT expandability hypothesis has relevant clinical implications. It explains, for example, the metabolic pattern usually observed in patients with lipodystrophies. These genetic diseases are characterized by different degrees of incapacity to expand subcutaneous adipose tissue (SAT) and increased ectopic fat in muscle [28], liver [29] and pancreas [30]. Consequently, patients with lipodystrophies have severe IR, which can lead to MetS, NAFLD and diabetes. The hypothesis also corroborates the action of thiazolidinediones (TZDs), insulin sensitizers approved to treat T2D. TZDs promote adipocyte differentiation of preadipocyte and mesenchymal stem cell lines, improving triglyceride storage capacity of the SAT [31] and increasing adiponectin levels [32], an adipokine with insulin sensitizer properties [33]. It suggests positive effects of TZDs on NAFLD, and this will be discussed below.

### Adipose tissue: a main culprit for metabolic health?

White adipose tissue is composed of SAT and visceral adipose tissue (VAT). SAT is the most appropriate local for fat storage due to its expandability and plasticity [34], while VAT is more associated with metabolic disorders. Nonetheless, some evidence corroborate that VAT may be a bystander and peripheral SAT may be of utmost importance for metabolic health [35, 36]. Impairment of peripheral fat storage capacity is etiological and genetically associated with IR and metabolic diseases [36] and support the AT expandability hypothesis. Moreover, a subgroup of normal weight metabolically unhealthy individuals is relatively frequent within the general population, which combined with the scarcity of leg fat [36], strongly suggests a polygenic lipodystrophy-like phenotype, with high risk for both NAFLD and cardiometabolic diseases.

Peripheral AT scarcity may partially explain NAFLD pathogenesis, but the AT insulin resistance per se may have a seminal role. Indeed, liver fat accumulation is strongly associated with diminishing AT insulin sensitivity, as evidenced by the negative correlation between liver fat content and the suppression of free fatty acids (FFAs) by insulin (r = − 0.38; p < 0.001), concordant with the lipotoxicity theory [37]. Moreover, hepatic IR become present early, with liver fat content ~ 1,5%, while muscle IR, high triglycerides and low HDL cholesterol become apparent when liver fat content reaches around 6,0%, suggesting that liver fat content works as a sensitive “barometer” for metabolic health [37]. In summary, AT may be pointed as a main culprit for NAFLD and metabolic disturbance [38].

### Genetic predisposition

Genome-wide association (GWA) studies found several genetic variants associated with NAFLD, which implies in variability on individual susceptibility to the disease [39]. PNPLA3 (encoding patatin-like phospholipase domain-containing protein 3) and TM6SF2 (encoding transmembrane 6 superfamily member 2) have more consistently demonstrated association with NAFLD prevalence and severity [39]. Interestingly, those genetic variants does not compromise metabolic profile [40]. For example, the strongest genetic risk for fatty liver to date, PNPLA3 variant rs738409-G, is associated with a neutral effect on lipids, and the TM6SF2 rs58542926-T, with a benign lipid profile [40]. This suggests that NAFL and/or NASH are not necessarily, by themselves, causal for cardiometabolic risk.

### The new liver-pancreas axis, diabetes and NAFLD

Insulin and glucagon are the main pancreatic hormones responsible for fuel homeostasis. They have a reciprocal pattern of release in response to glycemic oscillations [41]. Besides the well-known insulin relationship with liver glucose production and utilization, glucagon is an important player in liver glucose production and diabetes pathophysiology (briefly reviewed in [41]).

Knop et al. [42] and Holst et al. [43] proposed a new axis between the liver and the pancreatic α-cells. In physiological situation, glucagon increases the hepatic clearance of amino acids (AAs), so promoting ureagenesis. As AAs stimulate glucagon production and release by α-cells, the reduced circulating AAs relieves glucagon production, keeping them in balance. However, when the liver becomes greasy, there is a reduction in sensitivity to glucagon in the AAs metabolism (hepatic glucagon resistance), reducing ureagenesis and resulting in hyper-aminoacidemia. Consequently, increased AAs stimulate glucagon production to compensate for glucagon resistance, and a vicious cycle is installed. Liver-α-cell axis has been demonstrated in rodents [44, 45] and humans [42].

Increased fasting glucagon may precedes diabetes. Indeed, normal glucose tolerant obese patients have already fasting hyperglucagonemia [46], which is related to liver steatosis [47]. NAFLD patients have fasting hyperglucagonemia when compared to people without NAFLD regardless of diabetes presence [48]. In accordance, not only glucagon but also non-branched-chain AAs are increased in NAFLD patients and they correlate positively with each other [49].

Glucagon resistance is associated with glucagonotrophic AAs and this association is modified by increased liver fat content. Wewer Albrechtsen et al. [50] proposed a glucagon-alanine index [glucagon-alanine index = fasting plasma glucagon (pmol/L) × fasting plasma alanine (pmol/L)] as a marker of hepatic glucagon sensitivity in liver.

The other side of the liver-pancreas axis is suggested by the appearance of hepatic steatosis post-pancreatectomy (absence of α-cell) in dogs (reviewed in [42]) and humans [42, 51]. Indeed, knockout of glucagon receptors in mice lead to α-cell hyperplasia and steatosis [44, 45]. Additionally, Guzman et al. [52] showed a significant increase in liver fat content following the use of a new glucagon receptor antagonist in patients with T2D in comparison to sitagliptin and placebo (3,7% and 4,4% increment, respectively) [52].

We highlight that hepatic IR and glucagon resistance at the AAs metabolism can putatively contribute to T2D development in NAFLD patients [53], and it might have clinical and therapeutic implications. Treatments targeting weight loss and/or reducing liver fat content may restore the physiology of the liver-pancreas axis, so decreasing IR and glucagon levels, and possibly mitigating the increase in hepatic glucose production. Therefore, these may contribute to prevent T2D development or worsening [53].

### The multi-hit hypothesis

**Fig. 1:**
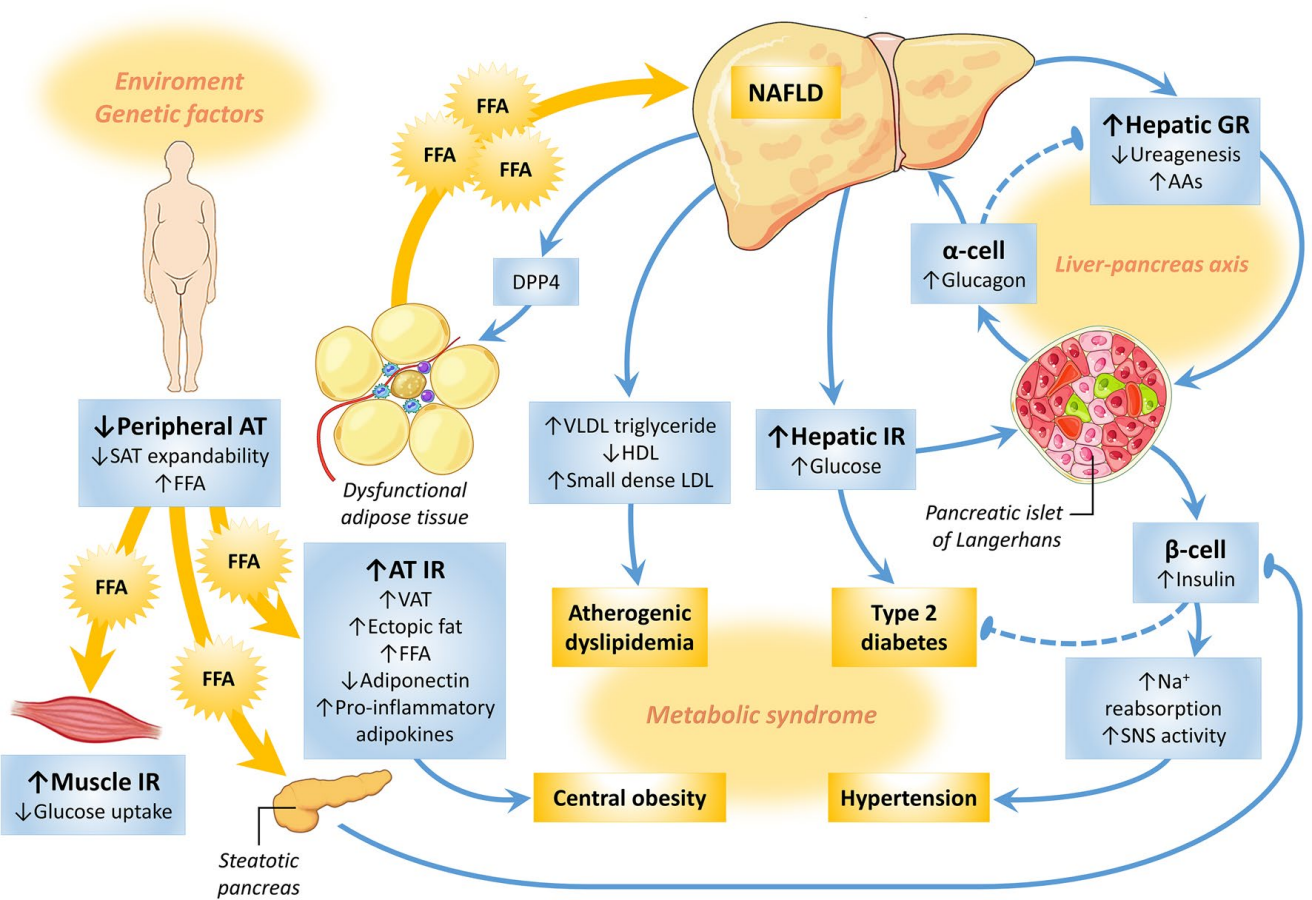
Pathophysiology of NAFLD as a continuum from obesity to metabolic syndrome and diabetes. Environmental factors affect the expression of genes, inducing weight gain. When the capacity of expansion of subcutaneous adipose tissue (AT) is reached, an increased free fatty acids (FFAs) mobilization arises, resulting in visceral and ectopic fat deposition. One ectopic site is the muscle, where increased FFAs deposition promotes insulin resistance (IR), inhibiting insulin-mediated glucose uptake. On the other hand, AT insulin resistance facilitates lipolysis and increases the flux of FFAs to the liver, inducing hepatic IR and enhancing glucose production, *de novo* hepatic lipogenesis, VLDL release and atherogenic dyslipidemia. FFAs spill over into the pancreas, causing β-cell dysfunction by lipotoxicity, hyperglycemia and diabetes (the twin cycle hypothesis). Increased liver fat also promotes hepatic glucagon resistance (GR) over the amino acids (AAs) metabolism, reducing ureagenesis and resulting in hyper-aminoacidemia. Increased AAs stimulate glucagon production to compensate for hepatic GR, and a vicious cycle is installed (the liver-pancreas axis). This hyperglucagonemia also leads to an increased hepatic glucose release. The globally IR state results in hyperinsulinemia, which may enhance sodium reabsorption and increase sympathetic nervous system activity, contributing to the hypertension. Inflamed dysfunctional AT becomes more insulin resistant and releases pro-inflammatory adipokines, while decreases anti-inflammatory adiponectin. In the liver, triglycerides and toxic metabolites induce lipotoxicity, mitochondrial dysfunction and endoplasmic reticulum stress, leading to hepatocyte damage, apoptosis and fibrosis. These dysfunctional hepatocytes synthesize and secret the dipeptidyl peptidase 4 (DPP4), which promotes inflammation of AT macrophages and more IR. *AAs* amino acids, *AT* adipose tissue, *DPP4* dipeptidyl peptidase 4, *FFA* free fatty acid, *GR* glucagon resistance, *HDL* high-density lipoprotein, *IR* insulin resistance, *LDL* low-density lipoprotein, *NAFLD* nonalcoholic fatty liver disease, *SAT* subcutaneous adipose tissue, *SNS* sympathetic nervous system, *VAT* visceral adipose tissue, *VLDL* very low-density lipoprotein. Pointed arrows indicate stimulation or enhancement, while blunt ends indicate inhibition or repression. Dashed arrows indicate progressive reduction in a pathway

Pathophysiology of NAFLD was originally suggested by the “two-hit hypothesis”. Fat accumulation in liver promotes IR (“first hit”), which in turn triggers inflammatory mechanisms and fibrosis (“second hit”). The “multi-hit hypothesis,” however, seems to be more complete, considering that environmental influences can affect the expression of genes, inducing weight gain, increased FFAs mobilization, ectopic fat deposition and IR [1] (Fig. 1). IR is a major factor in the genesis of NASH [37, 54], since it facilitates lipolysis, increasing the flux of FFAs to the liver and hepatic lipogenesis de novo. Inflamed dysfunctional AT releases adipokines and inflammatory cytokines as IL-6 and TNFα-1, while decreases anti-inflammatory adiponectin. In the liver, triglycerides and toxic metabolites induces lipotoxicity, mitochondrial dysfunction and endoplasmic reticulum stress, leading to hepatocyte damage, apoptosis and fibrosis [1].

## NAFLD diagnosis

NASH is characterized when, in addition to steatosis, there is evidence of lobular inflammation and hepatocyte ballooning with or without perisinusoidal fibrosis on the liver histology [5].

This could be considered the first gap of the NAFLD management. Even consisting the “gold standard” method for NASH diagnosis (and differentiation of NAFL), liver biopsy has some limitations related to invasiveness, patient discomfort, sampling variability and pathologist experience. Additionally, its cost-effectiveness needs to be demonstrated since approved NASH-specific therapies are currently not available [55].

### Liver biopsy

The American Association for the Study of Liver Diseases (AASLD) [5] recommends biopsy for patients with NAFLD who are at increased risk of NASH and/or advanced fibrosis, specifically when coexisting etiologies for hepatic steatosis and the presence or severity of any other liver diseases cannot be excluded without the biopsy.

Classically, some patients can be considered high-risk for NASH, including those with MetS, elevated aminotransferases (particularly with an elevated ALT/AST ratio), older age (> 60 years) and Hispanic ethnicity [55]. Important the most (as risk marker) is the number of MetS components (Table 2), reinforcing the idea of a continuous disease.

The pathology should be descriptive, including a distinction between NAFL, NAFL with inflammation and NASH (steatosis with lobular and portal inflammation and hepatocellular ballooning), and defining the presence or absence of fibrosis [5]. For clinical purposes, the description of severity (mild, moderate, severe) is indicated, as well as the use of specific scoring systems such as NAFLD Activity score (NAS) [56] and/or Steatosis Activity Fibrosis (SAF) [57].

Although liver biopsy is considered very useful in differentiating NASH from other diseases, most of the patients with NAFL will not progress to NASH and advanced fibrosis. Moreover, the routine screening for NAFLD with liver biopsy is unfeasible for a great number of high-risk patients, and this invasive procedure has several drawbacks, such as sampling error, high cost, inter- and intra-observer variability and risk of complications [58]. In this context, non-invasive methods could be performed for the detection and follow up of the patients.

### Non-invasive evaluation tests

#### Imaging techniques

##### Ultrasonography

In clinical practice, US is the first-line imaging exam used for the diagnosis of hepatic steatosis because its wide availability and lower-cost. A meta-analysis of 49 studies demonstrated an 84% of sensitivity and 93,6% of specificity for the detection of moderate-severe fatty liver when compared to histology (gold standard). The area under the curve (AUC) was 0.93 (95%CI 0.91–0.95) [59]. Despite that, biopsy-controlled study involving patients with NASH showed that the sensibility drops in initial steatosis, and a relevant number of patients can be missed when the US is used to detect 5–20% of fat liver disease [60].

##### Computed tomography

Steatosis may be detected on non-contrast CT, but due its lower sensitivity and exposure to radiation, it is less useful than US as a screening test [59].

##### Transient elastography (TE)

A best-validated non-invasive method for fibrosis evaluation is TE using US (e.g., FibroScan). It consists on measuring steatosis by reporting the loss of ultrasound signal through the liver parenchyma, which is reported as the controlled attenuation parameter (CAP) [61].

In a meta-analysis, CAP had a sensitivity and specificity of 78% and 79%, respectively, for detecting S1 steatosis [61]. CAP is less robust, however, in discriminating between steatosis grades, with an AUC of 0.73 and 0.70 for distinguishing S3 vs. S0–2 and S2–3 vs. S0–1, respectively [62].

The liver stiffness measurement (LSM), expressed in kilopascals (kPa) is another parameter of TE to measure shear wave’s velocity or liver fibrosis. It is of interest to exclude advanced fibrosis. In a cohort of 761 NAFLD patients, for instance, at a cutoff < 8 kPa, TE had a 94–100% negative predictive value [63].

##### Magnetic resonance imaging (MRI)

MRI has a better sensitivity for the evaluation of hepatic steatosis (with 92–100% sensitivity, 92–97% specificity) than US, but it is significantly more expensive. Magnetic resonance spectroscopy (MRI-S) and magnetic resonance imaging-estimated proton density fat fraction (MRI-PDFF) quantify steatosis. Although highly accurate, MRI-S only measures fat in small regions of interest, while MRI-PDFF allows mapping of the entire liver [60]. None of these imaging modalities can differentiate NAFL from NASH, and they have limited ability to discern those patients with advanced fibrosis.

On the other hand, MRI with elastography (MRE) is a better method for identifying degrees of fibrosis in patients with NAFLD. In a prospective cohort of 117 patients with biopsy-proven NAFLD, MRE showed a high diagnostic accuracy, with AUCs of 0.84 for the detection any fibrosis and 0.92 for advanced fibrosis. The optimal cutoff for advanced fibrosis was 3.64 kPa [64].

In another meta-analysis based on 5 studies and 628 NAFLD patients, the pooled AUC of MRE for advanced fibrosis was 0.96, showing the highest diagnostic accuracy for staging fibrosis in comparison to TE and indexes like Fibrosis-4 index (FIB-4) and NFS [65].

#### Scoring systems for estimative of steatosis or fibrosis

Several non-invasive models of blood biochemical markers or indexes have been proposed to estimate steatosis or fibrosis in NAFLD (Table 3). They are usually based on variables and calculated with formulas previously published. Among the scoring systems, FIB-4 index and NFS have been mostly studied and validated for estimative of fibrosis [66, 67]. They may be useful for excluding fibrosis, with a 97% specificity for stages F3 and F4 of fibrosis when NFS > 0.675 and FIB-4 index > 3.25 [68]. For estimating steatosis degree, WC and MetS are parameters used in Fatty Liver index and NAFLD Liver Fat score, respectively, reinforcing the continuous nature of the disease [69, 70].

**Table 3:** Scoring systems for estimative of steatosis or fibrosis in patients with nonalcoholic fatty liver disease. Adapted from [66–73]

Components	Steatosis			Fibrosis			
	Fatty liver index	NAFLD liver fat score	Hepatic steatosis index	BARD score	APRI	FIB-4 index	NFS
Age						X	X
Sex							X
BMI	X	X	X	X			X
Glycemia (or T2D diagnosis)		X	X	X			X
Platelet count					X	X	X
Albumin							X
AST		X	X	X	X	X	X
ALT		X	X	X		X	X
GGT	X						
Triglycerides	X						
WC	X						
MetS and insulin		X					

*ALT* alanine aminotransferase, *APRI* aspartate aminotransferase to platelet ratio index, *AST* aspartate aminotransferase, *BARD* body mass index, AST-to-ALT ratio, diabetes, *BMI* body mass index, *FIB-4* Fibrosis-4 index, *GGT* gamma-glutamyl transferase, *MetS* metabolic syndrome, *NAFLD* nonalcoholic fatty liver disease, *NFS* NAFLD Fibrosis score, *T2D* type 2 diabetes, *WC* waist circumference

### Multistep approach for NAFLD diagnosis and follow-up

Screening and diagnosing NAFLD is a multistep process. All patients at high risk (i.e., patients with obesity and/or MetS or T2D) might be targeted promptly for ruling out NAFL/NASH and fibrosis. Considering the increasing incidence of NAFLD and the inherent limitations of liver biopsy, we suggest a preferentially non-invasive approach.

The initial screening can be made with NAFLD liver fat score and Fatty liver index, both estimated from routinely available clinical and laboratory data. They have previously been correlated with liver fat content [69, 70] and may help in patient selection for conventional US, which is the currently recommended method to detect steatosis by the guidelines [5, 6].

Once steatosis is confirmed, TE quantification of steatosis could be provided with the CAP parameter. The steatosis degree can be graded as mild, moderate and severe [61]. Importantly, some clinical variables as race, BMI, and T2D are known to affect this image-based method and further adjustments are needed to improve its accuracy [74]. In addition, TE is a valuable tool for detection of fibrosis, an important prognostic marker of liver disease. The LSM parameter of TE significantly increases according to fibrosis stage, discriminating significant (F2–F4) and severe (F3–F4) fibrosis [63]. At this point, the use of non-invasive simple indexes as FIB-4, APRI and NFS can be associated and offers a good performance for ruling out or staging fibrosis [65]. Moreover, these indexes are the most consistent for identifying fibrosis progression or regression before and after an intervention [75].

Methods based on MRI, including MRE, are accurate for liver fat quantification, and promising to detect changes in fibrosis stage during the follow-up. The high cost and lack of availability are limitations of these methods. MRE may be recommended in selected cases, when the TE has lower accuracy, for instance in patients with ascites or severe obesity [74].

Despite the limitations of liver biopsy, we highlight it is the gold-standard method for NASH and fibrosis evaluation, and may be considered in selected cases, such as suspicion of other liver disease [56, 57].

## NAFLD treatment

### Treating obesity in patients with NAFLD

Excess body weight is an important cornerstone of NAFLD’s pathophysiology, and it is also a critical determinant of adverse clinical outcomes [76]. The new MAFLD definition reinforces this concept, since it is based on the presence of histological, imaging or blood biomarker evidence of hepatic steatosis in combination to overweight/obesity [8].

Weight loss has the strongest capacity to induce histological improvement in NASH. The amount of weight loss can determine NAFLD outcomes. Even modest weight loss of ≥ 3% can improve steatosis, while at least 5% weight loss is needed to improve inflammation and hepatic histology [77] and to stabilize fibrosis [78–80]. Seven percent or more of weight loss resolves NASH in 65–90% of patients and improves the NAS [78–80]. Ten percent or more, can improve fibrosis, leading to fibrosis regression in 45% of patients [77, 79].

There is a dose–response between weight loss and the magnitude of histological improvement and, according to the NAFLD guidelines, a 7–10% weight loss is the primary target of most interventions [5, 6].

#### Diet and exercise

The dietary management in NAFLD should focus on caloric restriction, aiming to induce 0.5–1 kg/week of weight loss. In general, a low-calorie diet should have 50–60% of caloric intake from carbohydrates and 20–25% from lipids [81, 82]. Saturated fats should account for < 10% of total fat intake [81, 82].

There is lack of evidence to recommend polyunsaturated fatty acids (PUFA) supplementation in patients with NAFLD. Despite some evidence of liver fat improvement on imaging, it failed to show significant histological improvement [83–90]. Furthermore, high meal frequency can increase the amount of intrahepatic fat and abdominal fat independent of caloric content and body weight gain [81, 82]. Therefore, snacking should be avoided in patients with NAFLD [82].

Very low-calorie diets (500–800 kcal/day) have low long-term adherence and are not recommended [81, 82]. The Mediterranean diet, which is rich in monounsaturated fatty acids, PUFA and polyphenols, showed improvement on insulin sensitivity and hepatic steatosis. According to many medical societies, Mediterranean diet is an eating pattern of choice in individuals with NAFLD [6, 91–94].

In general population with obesity, high protein diets may be an option to weight loss and maintenance. A high-protein, hypocaloric and high-fiber diet has shown a significant reduction of liver fat content and LSM, a surrogate marker of liver fibrosis [95–97]. Overall, these specific diets may be efficient methods of reducing caloric intake and have shown promising results in observational studies and small randomized controlled trials (RCTs) [81, 82]. On the other hand, further studies demonstrating histological improvement in NAFLD are needed. Type of diet and quantity of kcal restriction per day should be individualized, based on comorbidities and patient’s preferences.

If diet and exercise fail to achieve the targets in individuals with NAFLD, the addition of pharmacotherapy is recommended in individuals with BMI ≥ 27 kg/m^2^, since NAFLD is an obesity-related comorbidity [98–100]. Some of the approved antiobesity medications were investigated in NAFLD patients, including liraglutide, a glucagon-like peptide-1 receptor agonist (GLP-1RA), and orlistat.

#### GLP-1RAs

GLP-1RAs are very promise drugs for treatment of NAFLD. Acting through many mechanisms, these agents induce a dose-dependent weight loss, which probably implies improvement in fatty liver. Moreover, there is in vitro evidence that GLP-1 receptor is present on human hepatocytes and its activation by exendin-4 has been shown to reduce hepatocyte steatosis [101]. Additionally, GLP-1RAs can improve hepatic and adipose tissue IR and lipid metabolism, and decrease de novo lipogenesis, AT lipolysis, hepatic glucose production and oxidative stress [102, 103].

Apart from liraglutide, no other GLP-1RA is currently approved for the treatment of obesity. Notwithstanding, all GLP-1RAs slow gastric emptying, decrease appetite and increase postprandial satiety and fullness, beyond their insulin-stimulating and glucagon-inhibiting effects [104].

Weight loss is one of the mechanisms that could support GLP-1RAs benefits to NAFLD individuals. Liraglutide 3.0 mg/day has proven to induce 8.0 ± 6.7% of weight loss [105] and 63% median rate of achieving at least 5% weight loss [106]. Liraglutide also appears to decrease metabolic dysfunction, IR and lipotoxicity [102]. Therefore, some of the beneficial effects of liraglutide on NAFLD could be independent of weight loss. In rodents, liraglutide prevented the development of NAFLD and attenuated the expression of pro-inflammatory cytokines [107–110].

Some clinical trials evaluating different doses of liraglutide showed positive results on NAFLD, and the majority were performed in people with T2D (as discussed in the respective section below). In a 6-month RCT, liraglutide (3 mg/day) was shown to be similarly effective as the combination of diet and aerobic exercise in reducing weight, liver fat content (assessed by MRI) and ALT in obese NAFLD patients [111]. Additional larger studies using histological endpoints are needed before liraglutide can be recommended for treatment of NAFLD in patients with obesity.

Very recently, the result of a phase 2 trial (NCT02970942) evaluating the efficacy and safety of three different doses of semaglutide vs. placebo in NASH was announced by its fabricant [112]. In this press release, semaglutide was superior to placebo in attaining the primary endpoint of resolution of NASH and no worsening in liver fibrosis, and it is now being evaluated for further clinical trial development [112].

#### Orlistat

Orlistat promotes weight loss by inhibiting gastrointestinal and pancreatic lipases, thus preventing the absorption of approximately one-third of dietary triglycerides. It is associated with an estimated 44% median rate of achieving at least 5% weight loss [106]. The excess weight loss with orlistat compared to placebo (i.e., weighted mean difference for the drug-to-placebo comparison) was 2.6 kg (95% CI 2.3–2.9 kg) [106], which is considered a mild effect.

Five studies have investigated the effects of orlistat on liver endpoints [113–117]. All studies showed improvement in liver fat content, as well as levels of ALT and AST, and three studies evidenced improvement in histopathology [113, 114, 117], but these changes were not superior to other treatments such as lifestyle, sibutramine or even placebo [113–117].

Only a 24-week double-blinded, RCT with orlistat (n = 52) [117] assessed histological endpoints. There was a significant decrease in serum transaminases and a reversal of liver fat content (assessed by US), but no statistically significant difference in histological improvement in comparison to placebo [117]. Therefore, orlistat may have benefit for NAFLD as it induces weight loss, but there is lack of evidence that it is superior to other weight loss therapies or that it brings beneficial effects on the liver independent of weight reduction.

#### Bariatric surgery

If the combination of lifestyle modification and pharmacotherapy also fails in patients with NAFLD, then bariatric surgery should be considered in selected individuals with BMI ≥ 35 kg/m^2^, since NAFLD is an obesity-related comorbidity [98–100].

The effects of weight loss surgery on NAFLD, including sleeve gastrectomy (SG), Roux-en-Y gastric bypass (RYGB), and adjustable gastric banding (AGB), have been described. In a study comparing RYGB with AGB (n = 1236), NAFLD improved with both surgeries. However, RYGB induced more weight loss (26% vs. 21%) and had a better effect on NAFLD, despite the greater baseline BMI and the more severe NAFLD when compared to AGB group at 1 and 5 years [118]. A prospective observational study (n = 52) evaluating laparoscopic SG evidenced that 90% of subjects with baseline NAFLD (assessed by US) achieved its resolution on follow-up, and it was correlated with improvement in HDL cholesterol levels [119].

In a secondary analysis of an RCT, 72 patients who underwent SG or RYGB were identified with histological NAFLD using intraoperative liver biopsies [120, 121]. Those that underwent SG (n = 36) had significant improvements in AST, ALT and GGT at 12 months, which may indicate a greater benefit on liver fat and/or inflammation for SG, though follow-up histology was not performed [120, 121].

A recent meta-analysis of 21 studies, enrolling 2374 patients, assessed the resolution of NAFLD after bariatric surgery [122]. A high proportion (88%) of the subjects improved steatosis and steatohepatitis, and 30% improved or resolved liver fibrosis. RYGB had a greater impact on NAFLD histology when compared with other procedures [122]. Although additional data is needed to assess the optimal surgical strategies to improve NAFLD and to determine its cost-effectiveness, the available evidence to date suggests that bariatric surgery could be considered as a potential treatment for NAFLD.

### Treating NAFLD in patients with MetS

#### Metformin

Several trials evaluated the effect of metformin on treatment of NAFLD, but none of them was specifically designed to evaluate patients with MetS. A recent systematic review with 6 RCTs included 573 patients, most of them without diabetes (> 90%), with a mean BMI 30 ± 2.5 kg/m^2^, who were treated for a median of 9 months [123]. Among the four RCTs including adult patients with NAFLD confirmed by biopsy, small benefits were observed on liver steatosis and inflammation, but not on fibrosis [123]. A significant reduction was observed in serum aminotransferase levels (specially ALT), but this effect was not confirmed in another series [124, 125].

In the TONIC trial, the use of metformin failed to reduce ALT levels and improve liver histology compared with placebo in 173 children or adolescents with biopsy-proven NAFLD and without diabetes [125]. Similarly, there is no confirmed benefit for the use of metformin on liver disease for adults with NASH with MetS [123].

#### Pioglitazone

Pioglitazone, a thiazolidinedione (TZD) insulin sensitizer acting through an agonist effect on the peroxisome proliferator-activated receptor gamma (PPAR-γ) [31], have shown some benefit in RCTs in patients without diabetes. In the PIVENS trial [126], 247 patients with NASH and without diabetes were randomized to receive treatment with placebo, pioglitazone (30 mg/day) or vitamin E (Vit-E, 800 UI/day) for 2 years [126]. Compared to placebo, the rate of improvement in NASH with pioglitazone was not significant (34% and 19%, respectively; p = 0.04). Due to two primary comparisons, a *p* value less than 0.025 was considered statistically significant. Notwithstanding, there were significant improvements in steatosis (p < 0.001) and lobular inflammation (p = 0.004). If a finding of no worsening of hepatocellular ballooning was used, however, NASH improvement with pioglitazone became significant (48% vs. 25%, p = 0.003). Notably, the resolution of NASH, a key secondary endpoint, was achieved with statistical significance in more patients using pioglitazone, when compared to placebo (47% vs. 21%, p = 0.001) [126].

Another RCT with 74 patients with biopsy-proven NASH and without T2D showed reduction in liver fat content and liver fibrosis after 12 months of use of pioglitazone 30 mg when compared to placebo [127].

The benefic effect of TZDs in the treatment of NASH were corroborated in a systematic review with 8 RCTs using pioglitazone (06 trials) or rosiglitazone (02 trials) to specifically treat NAFLD or NASH including 828 individuals, most of whom (85%) did not have diabetes and were treated for a median of 12 months. In comparison to placebo or reference therapy, both TZDs significantly improved liver fat content and NASH. A significant reduction of serum aminotransferase levels was observed in most patients treated with TZDs, when compared to placebo or reference therapy [123].

A previous meta-analysis demonstrated that in 4 RCT in patients without diabetes, the use of TZDs was associated with improvement in advanced fibrosis (OR 2.95, 95% CI 1.04–10.90, p = 0.02, I^2^ = 0%), improvement in fibrosis of any stage (OR 1.76, 95% CI 1.02–3.03, p = 0.02, I^2^ = 0%) and NASH resolution (OR 3.40, 95% CI 1.95–5.93, p < 0.001, I^2^ = 0%). The effects were mainly accounted for the use of pioglitazone [128].

Taken together, these results demonstrate the benefic effect of pioglitazone in patients with MetS and biopsy-proven NASH and fibrosis. It is important to remember, however, the side effects of TZDs, such as weight gain, fluid retention, risk of congestive heart failure, decrease of bone mineral density and a higher risk for fractures [31]. So, the use of pioglitazone may be considered after discussion of risks and benefits with each patient [5].

#### Vitamin E

The use of Vit-E, a fat-soluble vitamin with antioxidant properties, has been investigated in different clinical trials, particularly due to its potential of improvement in steatosis, inflammation and resolution of steatohepatitis in adults with NASH. In the PIVENS trial, the use of Vit-E therapy (800 IU/day) for 2 years achieved the primary endpoint improvement in the NAS by 2 or more points and no increase in fibrosis when compared to placebo (43% vs. 19%, p < 0.001; number needed to treat = 4.4) [126].

Some meta-analyses raised concern about long term safety of high doses of Vit-E (> 800 IU/day) analyzing all-cause mortality, prostate cancer and hemorrhagic stroke [129, 130]. On the other hand, another large meta-analysis with 57 trials reported no effect on overall mortality with doses up to 5500 IU/day of Vit-E [131]. In summary, Vit-E supplementation may be considered for biopsy-proven NASH in patients without diabetes, after careful discussion of risks and benefits of the therapy [5].

#### Selonsertib and other anti-inflammatory drugs

The understanding of pathogenic mechanisms involving NAFLD progression, such as chronic inflammation and fibrogenesis in the liver, brought some speculation about anti-inflammatory and antifibrotic therapies [132]. Selonsertib, an orally bioavailable inhibitor of apoptosis signal-regulating kinase 1, and simtuzumab, a humanized monoclonal antibody designed for the treatment of fibrosis, are examples of these agents [132]. A phase 2 trial evaluated the use of selonsertib alone or in combination with simtuzumab in patients with NASH and moderate to severe liver fibrosis (stage 2 or 3). The trial was terminated after 96 weeks due to lack of efficacy [132].

### Treating NAFLD in patients with diabetes

Because NAFLD and T2D have the same pathophysiological origin, it is reasonable to suppose that drugs to treat T2D, and the metabolic surgery, have the potential to also treat NAFLD. According to Diabetes Guideline of the Brazilian Diabetes Society [31], beyond insulin, there are eight classes of antidiabetic agents, and all of them were tested in the treatment of NAFLD. A brief review of these classes of drugs and their mechanisms of action can be found in Table 4.

**Table 4:** Classes of antidiabetic agents and their respective mechanisms of action. Adapted from [31]

Class of antidiabetic agent	Mechanism of action
Metformin	Reduction in hepatic glucose production and mild insulin sensitizing action in the liver
Thiazolidinediones	Increase insulin sensitivity in muscle and adipocyte (insulin sensitizers)
DPP4 inhibitors (gliptins)	Increase in GLP-1 levels, enhancing the glucose-dependent synthesis and secretion of insulin, in addition to glucagon reduction
GLP-1RAs	Enhancement of the glucose-dependent synthesis and secretion of insulin, in addition to glucagon reduction, delayed gastric emptying and promotion of satiety, resulting in weight loss
SGLT2 inhibitors	Inhibition of glucose and sodium reabsorption in the proximal tubule of the renal glomerulus, resulting in glycosuria and weight loss
Sulfonylureas	Glucose-independent secretion of insulin (secretagogue)
Glinides	Glucose-independent secretion of insulin (secretagogue)
α-Glucosidase inhibitors	Delay of intestinal absorption of carbohydrates

*DPP4* dipeptidyl peptidase 4, *GLP-1* glucagon-like peptide-1, *GLP-1RAs* glucagon-like peptide-1 receptor agonists, *SGLT2* sodium-glucose cotransporter-2

#### Metformin

According to a systematic review evaluating antidiabetic agents for NAFLD [133], there are five RCTs with metformin in this context, none of them exclusive in patients with T2D. In the study with the highest proportion of patients with T2D (27.3%), treatment with metformin (2500 mg/day or 3000 mg/day, if body weight was > 90 kg) or placebo for 6 months did not resulted in significant differences for changes in liver steatosis (assessed either histologically or by CT), NAS or liver transaminases [124].

In summary, metformin did not substantially impact NAFLD [133]. Nevertheless, it does not mean that it is useless for NASH complications. In a nationwide case–control study in Taiwanese population, metformin use was associated with a decrease in the risk of hepatocellular cancer in a dose-dependent manner [134]. Each incremental year of metformin resulted in 7% reduction in the risk of hepatocellular cancer in patients with T2D followed up for 12–16 years (adjusted OR 0.93, 95% CI 0.91–0.94, p < 0.0001) [134]. In hepatoma cell lines, metformin inhibits cell growth through cell cycle G0/G1 arrest, an effect partially attributed to the activation of adenosine monophosphate-activated protein kinase (AMPK) pathway and its upstream liver kinase B1 (LKB1), with antiproliferative effects [134].

#### Thiazolidinediones

TZDs are the glucose-lowering agents most extensively explored on NAFLD. Considering the pathophysiology of NAFLD, TZDs seem a reliable therapeutic option for patients with T2D.

In a proof of concept study, Ravikumar et al. [135], treated 10 T2D patients with 30 mg/day for 16 weeks and demonstrated an approximately 50% reduction in liver fat content (measured by MRI-S), what significantly correlated with decrease in fasting and postprandial endogenous glucose production. Interestingly, they also observed a decrease in fasting and postprandial glucagonemia. We speculate that pioglitazone, by reducing liver fat, favorably interfered with the new described liver-α-cell axis [42, 43, 47–53], reducing glucagon and contributing to glucose homeostasis.

A systematic review and meta-analysis of six RCTs (n = 332) evaluated the effect of TZDs (pioglitazone, rosiglitazone and troglitazone) vs. placebo or sulfonylureas on NAFLD in patients with T2D [136]. TZDs significantly decreased 6,6% of liver fat content (95% CI − 12.56 to − 0.96, p = 0.022, I^2^ = 0%). In an independent trial in patients with prediabetes or T2D and NASH, pioglitazone (45 mg/day) also decreased liver fat content by 54% in comparison to placebo (p < 0.001) [137].

Two trials that conducted liver biopsy in patients with prediabetes or T2D and NASH suggested that pioglitazone (45 mg/day) improved liver histology of steatosis, ballooning necrosis and inflammation, compared with placebo [137, 138]. Only one evidenced a significant improvement in fibrosis score with pioglitazone for 18 months (p = 0.039) [138]. TZDs also seem to be effective in improving ALT [136].

Interestingly, in a double-blinded, proof-of-concept RCT, Bril et al. [139] tested a combination of pioglitazone (45 mg/day) plus Vit-E (400 UI BID) vs. placebo or Vit-E in T2D patients with biopsy-proven NASH (n = 105). The primary histological endpoint of at least 2 points reduction in NAS, without any worsening in fibrosis, was achieved in more patients in combination therapy as compared to placebo (54% vs. 19%, p = 0.003), but not in the Vit-E group (31% vs. 19%, p = 0.26). While both groups achieved improvement in NASH (combination 43% vs. 12%, p = 0.005, and Vit-E alone: 33% vs. 12%, p = 0.04), ballooning and inflammation improved in combination group only [139]. Taken together, pioglitazone seems to be the best option for NASH therapy, at least in T2D patients, and it is incorporated in AASLD guidance for NASH therapy in patients with or without T2D [5].

Currently, pioglitazone is the only TZD available in Brazil. No dose adjustment is necessary in patients with mild-moderate liver failure, but pioglitazone should be avoided in those with severe liver dysfunction [31].

#### Dipeptidyl peptidase 4 (DPP4) inhibitors (gliptins)

DPP4, also known as adenosine deaminase binding protein or cluster of differentiation 26 (CD26), is a serine exopeptidase able to inactivate various oligopeptides through the removal of N-terminal dipeptides [140]. The activity of DPP4 seems to be increased in patients with T2D and there are a fair number of in vitro and in vivo studies demonstrating that this enzyme can interact with proinflammatory pathways [140]. DPP4 is also an hepatokine [141], and chronic liver diseases, including hepatitis C, hepatitis B, NAFLD and hepatocellular carcinoma, have been related to elevated levels of this enzyme [142].

There is a direct association between DPP4 activity and IR in humans [143], and evidence that obesity in mice stimulates hepatocytes to synthesize and secret DPP4, which acts with plasma factor Xa to promote inflammation of AT macrophages and IR [144] (Fig. 1). Curiously, silencing expression of DPP4 on hepatocytes suppressed inflammation of VAT and IR, but this effect did not occur with sitagliptin, an orally administered DPP4 inhibitor [144]. Once there are differences in the way in which gliptins interact with the DPP4, it may impact on the DPP4 inhibitors’ possible ability to mitigate inflammation and IR promoted by the hepatocyte-secreted DPP4 [141].

Clinical trials evaluating DPP4 inhibitors to NAFLD in people with T2D are scarce and conflicting. In a study involving patients with T2D randomized to vildagliptin (50 mg twice a day) or placebo over 6 months, mean fasting liver fat content (assessed by MRI) decreased by 27% with vildagliptin, while there was no change in placebo group. ALT fell significantly in the vildagliptin group, and there was a correlation between the decrements in ALT and liver fat content (r = 0.83; p < 0.0001) [145]. On the other hand, an RCT conducted in Chinese patients with T2D and NAFLD evidenced no significant changes in the average AST and ALT during the 52-week follow-up in both the sitagliptin (50 or 100 mg/day) and diet plus exercise groups [146]. To the best of our knowledge, there were no studies with DPP4 inhibitors and biopsy confirmed NASH.

There are minimal pharmacokinetic changes for DPP4 inhibitors in patients with varying degrees of liver dysfunction, except for vildagliptin, which is not recommended in patients with ALT or AST levels > 2.5 to 3 times the upper limit of normal [31]. Overall, it seems that the effectiveness of gliptins to treat NAFLD, if any, appears to be limited in people with T2D.

#### GLP-1RAs

Most of the clinical studies evaluating the hepatic benefits of GLP-1RAs in people with T2D are limited to the short-acting subcutaneous agents liraglutide and exenatide. In an individual patient-level meta-analysis of more than 4000 patients with T2D, comparing 26 weeks of liraglutide (1.8 mg/day) vs. placebo, liraglutide significantly improved liver enzyme concentrations in a dose-dependent manner [147]. Furthermore, according to a recent systematic review [123], there are only four RCTs evaluating the effects of GLP-1RA on NAFLD that have included patients with T2D, and only one of them included subjects with biopsy-proven NASH [148].

The LEAN trial was a multicentre, double-blind, phase 2 RCT to assess liraglutide (1.8 mg/day) vs. placebo for 48 weeks in patients with biopsy-proven NASH (32.6% with T2D) [148]. The primary outcome measure was resolution of NASH with no worsening in fibrosis from baseline to end of treatment. Nine (39%) of 23 patients on liraglutide had resolution of NASH compared with two (9%) of 22 patients in the placebo group (RR 4.3, 95% CI 1.0–17.7, p = 0.019) [148]. Two (9%) of 23 patients in the liraglutide group vs. 8 (36%) of 22 patients in the placebo group had progression of fibrosis (RR 0.2, 95% CI 0.1–1.0, p = 0.04) [148].

In summary, data from RCTs evidences short-acting GLP-1RAs seem to reduce serum liver enzymes and improve hepatic steatosis, as detected by either imaging techniques or liver histology [123]. Additionally, data from a 104-week cardiovascular outcomes trial in T2D evidenced the long-acting semaglutide (0.5 or 1.0 mg/week) significantly reduced ALT and hsCRP in comparison to placebo [149].

If larger phase 3 trials will further confirm the promising findings of the LEAN trial, it is reasonable to hypothesize that GLP-1RAs will become a suitable treatment option in NAFLD patients, especially in those with T2D [123].

#### SGLT2 inhibitors

Recent emerging evidence of the use of SGLT2 inhibitors in patients with NAFLD and T2D is promising. These agents have shown to reduce body weight, decrease levels of serum transaminases and improve steatosis and liver histology [150]. Empagliflozin, dapagliflozin and canagliflozin are the SGLT2 inhibitors currently available in Brazil [31].

A systematic review [151] assessed the effect of SGLT2 inhibitors on liver enzymes in patients with T2D and NAFLD. Data from eight studies (04 RCTs and 04 observational studies) lasting at least 12 weeks were extracted. Almost all (seven) studies showed a significant decrease in ALT, and most of the studies evidenced reductions in AST and GGT levels [151]. SGLT2 inhibitors were associated with significant reduction in liver fat content, and among the three studies that evaluated indices of hepatic fibrosis, a significant improvement was evidenced in two of them [151].

In the real-world E-LIFT trial [152], fifty patients with NAFLD and T2D under standard treatment were randomly assigned to receive empagliflozin (10 mg/day) or keep standard treatment without empagliflozin for 20 weeks. Empagliflozin reduced liver fat content (assessed by MRI) and improved ALT levels, but not GGT and AST levels [152]. Furthermore, results from RCTs showed a high consistent reduction in aminotransferases with empagliflozin in individuals with T2D, in a pattern (reductions in ALT > AST) that is potentially consistent with a reduction in liver fat content [153]. These ALT reductions were largely independent of changes in weight or HbA1c [153].

A randomized, active-controlled, open-label trial evaluated the use of dapagliflozin (5 mg/day) vs. standard treatment without SGLT2 inhibitors for 24 weeks in patients with T2D and NAFLD. There were significant improvements in ALT, GGT and liver stiffness assessed by elastography in the dapagliflozin group [154]. Dapagliflozin also reduced hepatic steatosis and attenuated fibrosis in a subgroup of patients with significant liver fibrosis (liver stiffness measurement ≥ 8.0 kPa) [154]. Additionally, the EFFECT-II trial [155] investigated the effects of dapagliflozin (10 mg/day), omega-3, and a combination of both vs. placebo on liver fat content (assessed by MRI) in subjects with T2D and NAFLD for 12 weeks. All active treatments significantly reduced liver fat content from baseline, but only the combination treatment reduced liver fat content (p = 0.046) and total liver fat volume (p = 0.037) in comparison with placebo [155]. Dapagliflozin monotherapy, but not the combination, reduced the levels of hepatocyte injury biomarkers, including ALT, AST and GGT [155].

A systematic review and meta-analysis of RCTs evaluated the effects of canagliflozin (100 or 300 mg/day) on liver enzymes in patients with T2D [156]. Eleven studies placebo-controlled or active-controlled were selected (n = 6745). Canagliflozin significantly decreased serum concentrations of ALT, AST and GGT after 26 and 52 weeks, suggesting a protective effect on liver [156]. Additionally, in a prospective small uncontrolled study, nine patients with NAFLD and T2D were subjected to liver biopsies at baseline and after 24 weeks of treatment with canagliflozin (100 mg/day) [157]. There was histological improvement in all patients. Scores of steatosis, lobular inflammation, ballooning, and fibrosis stage decreased by 78%, 33%, 22% and 33% at 24 weeks compared to the pretreatment, respectively [157].

Despite the very promise preliminary results, more clinical trials assessing the effect of SGLT2 inhibitors on NAFLD in patients with T2D are warranted, especially those primarily aimed to investigate the impact in hepatic histological features.

#### Sulfonylurea and glinides

Sulfonylurea and glinides are hypoglycemic drugs used to treat T2D. They share similar mechanisms of action, through the sulfonylurea receptor (SUR) on the β-cell, then stimulating insulin release and improving glycemic control [31]. Despite no plausible explanation for benefits of these secretagogues in NAFLD treatment beyond improvement in diabetes control, sulfonylureas were active comparators in few studies evaluating other antidiabetic drugs in this context and, in general, seems to be inefficient or less efficient than these other drugs on liver fat [136, 158, 159].

There are two RCTs in patients with T2D, respectively evaluating the effects of a sulfonylurea (gliclazide) and a glinide (nateglinide) on NAFLD, to be highlighted. In a 24 weeks clinical trial, 87 subjects were randomized to receive gliclazide, metformin, or liraglutide for 24 weeks. Primary outcomes included liver fat content, assessed by US, and liver function. All treatment groups resulted in significant decreased of these outcomes, but gliclazide resulted in less improvement compared with liraglutide and metformin [159]. Moreover, a very small study consisting in 10 subjects on diet and exercise therapy for T2D and biopsy-proven NASH, randomly distributed to nateglinide (270 mg/day) or no additional treatment, evidenced significative improvements in ALT, abdominal US and CT imaging tests and liver histological findings with nateglinide for 18 months [160].

#### α-Glucosidase inhibitors

Inhibitors of the intestinal enzyme α-glucosidase reduce postprandial glycemia by decreasing glucose absorption. Acarbose is the only α-glucosidase inhibitor currently available in Brazil [31].

Data are very scarce on the effect of acarbose to treat NAFLD. Histological benefits were evidenced in two experimental studies combining acarbose and ezetimibe in animal models of IR [161, 162]. In a subgroup analysis of a small clinical trial, involving subjects with an elevated hepatic fat content assessed by MRI-S, liver fat content was reduced in 26% under acarbose treatment (300 mg/day) for 12 weeks [163]. Nonetheless, no definitive conclusion can be drawn from this first human data due to small observation sizes.

Despite limited evidence of acarbose for NAFLD, the results of a double-blind cross-over study evaluating the safety and efficacy of acarbose (300 mg/day) vs. placebo for 24 weeks suggest it may be used for the treatment of T2D in patients with well-compensated liver cirrhosis [164].

#### Metabolic surgery

Several gastrointestinal operations and bariatric procedures promote improvement (and even remission) of MetS [165] and T2D [166]. In the ensuing years, the concept of “metabolic surgery” or “diabetes surgery” has become widely recognized, and most major worldwide bariatric surgery societies have changed their names to include the word “metabolic” [166, 167]. According to a joint statement by international diabetes organizations [166], metabolic surgery should be recommended to treat T2D in patients with class III obesity (BMI ≥ 40 kg/m^2^) and in those with class II obesity (BMI 35–39.9 kg/m^2^) when hyperglycemia is inadequately controlled by lifestyle and optimal medical therapy. Surgery should also be considered for patients with T2D and BMI 30–34.9 kg/m^2^ if hyperglycemia is inadequately controlled despite optimal treatment with either oral or injectable medications [166]. These recommendations do not consider the presence of NAFLD.

While some authors argue that NAFLD should be considered a comorbidity that lowers the BMI threshold for metabolic surgery to 35 kg/m^2^ [168], the American Society for Metabolic and Bariatric Surgery recommends that surgical treatment should be offered as an option for suitable patients with BMI 30–35 kg/m^2^ and obesity related co-morbidities, including NAFLD, who do not achieve substantial, durable weight loss and co-morbidity improvement with reasonable nonsurgical methods [169].

To date, the only study evaluating NAFLD remission after metabolic surgery including patients with class I obesity was the one conducted by Berry et al. [170]. This retrospective cohort study included 252 patients with BMI 30–35 kg/m^2^ and at least one associated comorbidity, such as NAFLD (n = 69) and/or T2D (n = 10). Over 3 years of postoperative follow-up, NAFLD (assessed by US) remitted in 84.6%, and T2D remitted in 60% and improved in 40% [170]. It was not clear, however, how many patients had both conditions at baseline. Therefore, the role of metabolic surgery to treat NAFLD in patients with T2D and class I obesity remains uncertain.

Considering NAFLD as a continuum, its treatment interventions according to patients’ profile are summarized in Table 5.

**Table 5:** Summary of the interventions to treat NAFLD according to patients’ profile

Intervention	Obesity	MetS	T2D
Caloric restriction and exercise	Recommended (despite unavailable evidence of LHI)	Recommended (despite unavailable evidence of LHI)	Recommended (despite unavailable evidence of LHI)
Orlistat	Modest benefits related to weight loss	#	#
Bariatric/metabolic surgery	Some benefic effects (unavailable evidence of LHI)	#	#
Metformin	No confirmed benefit	No confirmed benefit	No substantial impact, but may prevent NASH complications
Pioglitazone	Benefic effects, including LHI May be considered for BPN	Benefic effects, including LHI May be considered for BPN	Recommended (benefic effects, including LHI)
Vitamin E	Benefic effects, including LHI May be considered for BPN	Benefic effects, including LHI May be considered for BPN	Limited evidence of benefits Consider in combination with pioglitazone
DPP4 inhibitors	#	#	Benefits, if any, appears to be limited
GLP-1RAs	Benefic effects with liraglutide (3 mg/day), similarly effective as structured lifestyle modification (unavailable evidence of LHI) Preliminary evidence of resolution of NASH and no worsening in liver fibrosis with semaglutide (press release)	#	Benefic effects with liraglutide (1.8 mg/day), including limited evidence of LHI Preliminary studies with semaglutide are promising
SGLT2 inhibitors	#	#	Despite the very promise preliminary results, there is still no evidence of LHI
Sulfonylureas	#	#	Benefits, if any, appears to be limited with gliclazide
Glinides	#	#	Poor evidence of LHI with nateglinide
Acarbose	#	#	Very scarce data

^#^Not specifically evaluated in this population

*BPN* biopsy-proven nonalcoholic steatohepatitis, *DPP4* dipeptidyl peptidase 4, *GLP-1RAs* glucagon-like peptide-1 receptor agonists, *LHI* liver histological improvement, *SGLT2* sodium-glucose cotransporter-2

For more details and references, please consult the respective section on this review

## Conclusions

NAFLD is metabolically related with AT insulin resistance, limited expandability, and dysfunctionality [37, 38]. A fatty liver is a main driver for a new recognized liver-pancreatic α-cell axis and increased glucagon [42, 43, 47–53], putatively contributing to diabetes pathophysiology.

Patients with obesity and/or MetS, with or without T2D, might be targeted promptly for ruling out NAFL/NASH and fibrosis [3, 5, 6].

Treatment of the NAFLD spectrum is better accomplished with lifestyle measures, what may be associated with some drugs [3, 5, 6]. Weight loss of 7%-10% or more may revert steatosis and NASH [5, 6]. Liraglutide 3 mg/day can be considered a valuable option to treat obesity and consequently ameliorate NAFLD [111]. Bariatric surgery should be considered for those with a BMI ≥ 35 kg/m^2^ [98–100, 166, 168].

Among several drugs herein discussed, pioglitazone is the only recommended in specialized societies guidelines for the treatment of NAFLD [3, 5, 6]. Vit-E has showed histological improvements in patients without diabetes [5, 126], but specific trials are warranted in T2D.

GLP-1RAs are a probable fruitful class of agents due to their weight loss effects as much as some metabolic driven actions [102, 103, 107–111, 123, 147–149]. SGLT2 inhibitors have demonstrated some benefits in surrogate endpoints but need histological data [150–157]. Combination therapy (e.g., pioglitazone plus GLP-1RA and/or SGLT2 inhibitor) has never been studied and it is an avenue to be explored.

## Data Availability

Not applicable.

## Abbreviations

AAs: Amino acids
AASLD: American Association for the Study of Liver Diseases
AGB: Adjustable gastric banding
ALT: Alanine aminotransferase
AMPK: Adenosine monophosphate-activated protein kinase
APRI: Aspartate aminotransferase to platelet ratio index
AST: Aspartate aminotransferase
AT: Adipose tissue
AUC: Area under the curve
BARD: Body mass index, AST-to-ALT ratio, diabetes
BID: *bis in die* (twice a day)
BMI: Body mass index
BPN: Biopsy-proven nonalcoholic steatohepatitis
CAP: Controlled attenuation parameter
CD26: Cluster of differentiation 26
CI: Confidence interval
CT: Computed tomography
DPP4: Dipeptidyl peptidase 4
FIB-4: Fibrosis-4 index
GGT: Gamma-glutamyl transferase
GLP-1: Glucagon-like peptide-1
GLP-1RA: Glucagon-like peptide-1 receptor agonist
GR: Glucagon resistance
HbA1c: Hemoglobin A1c
HDL: High-density lipoprotein
HOMA-IR: Homeostasis model assessment of insulin resistance
hsCRP: High-sensitivity C-reactive protein
IL-6: Interleukin-6
IR: Insulin resistance
kPa: Kilopascals
LDL: Low-density lipoprotein
LHI: Liver histological improvement
LKB1: Liver kinase B1
LSM: Liver stiffness measurement
MAFLD: Metabolic-associated fatty liver disease
MetS: Metabolic syndrome
MRE: Magnetic resonance imaging with elastography
MRI: Magnetic resonance imaging
MRI-PDFF: Magnetic resonance imaging-estimated proton density fat fraction
MRI-S: Magnetic resonance spectroscopy
NAFL: Nonalcoholic fatty liver
NAFLD: Nonalcoholic fatty liver disease
NAS: NAFLD Activity score
NASH: Nonalcoholic steatohepatitis
NFS: NAFLD Fibrosis score
NHANES: National Health and Nutrition Examination Survey
OR: Odds ratio
PPAR-γ: Peroxisome proliferator-activated receptor gamma
PUFA: Polyunsaturated fatty acid
RCT: Randomized clinical trial
RYGB: Roux-en-Y gastric bypass
SAF: Steatosis Activity Fibrosis
SAT: Subcutaneous adipose tissue
SG: Sleeve gastrectomy
SGLT2: Sodium-glucose cotransporter-2
SNS: Sympathetic nervous system
SUR: Sulfonylurea receptor
T2D: Type 2 diabetes
TE: Transient elastography
TNF-α1: Tumor necrosis factor alpha 1
TZD: Thiazolidinedione
US: Ultrasonography
VAT: Visceral adipose tissue
Vit-E: Vitamin E
VLDL: Very low-density lipoprotein
WC: Waist circumference
